# Ultra-simplified Single-Step Fabrication of Microstructured Optical Fiber

**DOI:** 10.1038/s41598-020-66632-3

**Published:** 2020-06-15

**Authors:** Cristiano M. B. Cordeiro, Alson K. L. Ng, Heike Ebendorff-Heidepriem

**Affiliations:** 1 “Gleb Wataghin” Institute of Physics, University of Campinas, Campinas, 13083-859 Brazil; 2 Institute of Photonics & Advanced Sensing, The University of Adelaide, Adelaide, SA5005 Australia; 3 ARC Centre of Excellence for Nanoscale BioPhotonics, Adelaide, SA 5005 Australia

**Keywords:** Fibre optics and optical communications, Optical sensors

## Abstract

Manufacturing optical fibers with a microstructured cross-section relies on the production of a fiber preform in a multiple-stage procedure, and drawing of the preform to fiber. These processes encompass the use of several dedicated and sophisticated equipment, including a fiber drawing tower. Here we demonstrate the use of a commercial table-top low-cost filament extruder to produce optical fibers with complex microstructure in a single step - from the pellets of the optical material directly to the final fiber. The process does not include the use of an optical fiber drawing tower and is time, electrical power, and floor space efficient. Different fiber geometries (hexagonal-lattice solid core, suspended core and hollow core) were successfully fabricated and their geometries evaluated. Air guidance in a wavelength range where the fiber material is opaque was shown in the hollow core fiber.

## Introduction

Optical fibers revolutionized the way we communicate, being responsible for most of the actual global data traffic. Today there are hundreds of millions of kilometers of optical fibers installed around the planet. The data traffic is doubling every two years what represents a 1000-fold increase in just 20 years.

Optical fibers had a significant development at the end of the 1990s when structures with an internal microstructure cross-section were proposed and developed. Pioneered by Philip Russell from Univ. of Bath (UK) and his research team, the development of photonic crystal fibers (PCF), or microstructured optical fibers (MOF), expanded and revolutionized the whole field of guided optics^1–3^. The presence of wavelength-scale structures with high index contrast (fiber material to air) opened the possibility to extensively control the optical properties of the fiber. Chromatic dispersion, modal area, cladding evanescent field, birefringence, and non-linearity, for example, can be very dependent on the specific holes distribution - size, shape, position^1,2^. Conventional optical fibers, on the other hand, have a small core/cladding index contrast, usually below 1%.

While fibers with a solid core and a holey cladding with a lower refractive index guide by total internal reflection as traditional optical fibers, hollow-core fibers (HCFs) enabled new guiding mechanisms. Complex cladding designs allow the guidance via photonic bandgap. Simpler structures provide low loss transmittance via inhibited coupling^4^ or anti-resonance^5^.

While most traditional fibers and MOFs are made of silica due to their remarkable optical and physical properties, fibers can also be made of polymers and non-silica glasses. In the early 2000´s microstructured polymer optical fibers were developed^6^ extending the application of conventional polymer fibers.

In all cases, optical fibers are usually drawn in a multiple-stage process whose primary step is the manufacture of an enlarged version of the fiber, the preform. Different approaches have been used to produce the macroscopic preform. Standard optical fibers rely on vapor deposition methods to produce low loss preforms. MOF preforms with their characteristic air holes array, on the other hand, have been produced with different techniques. Silica MOFs are typically made via the stack-and-draw technique^1^ where millimeter thick capillaries are manually stacked, forming the desired structure. This is a convenient and versatile procedure when tubes are widely available commercially such as for silica and, also, for some borosilicate glasses (e.g. Duran^7^). However, stacking is time-consuming. Soft-glass MOFs can also be produced with this procedure but with extra complexity due to the initial need to produce the tubes^8^.

Polymer MOFs have been manufactured by directly drilling the holes in the plastic rod^6^, a technique that has also been applied with glasses^9,10^. Like the stacking technique, drilling is limited to circular holes. It is also restricted to short preforms. An alternative is casting the fiber material in a pre-designed mold, a procedure used to form plastic^11^ and glass fibers^12^.

Soft glass and polymer preforms have also been prepared via billet extrusion, a direct and straightforward way to obtain structures with elaborate designs. Billet extrusion involves the preparation of a billet from the chosen optical material, heating the billet to reduce its viscosity (to typically 10^8^-10^10^ dPa⋅s^13^) and, with the help of a ram, forcing the materials through a die with the desired pattern^14^. The extrusion die comprises an initial section with holes to feed the material to be extruded and a posterior segment that has solid features for blocking the material flow in pre-defined regions, allowing the extrusion of a preform with a holey pattern. This technique was shown to be successful in producing high-quality MOFs from soft-glass^14^ (such as lead-silicate, tellurite, bismuth, fluoride, chalcogenide, phosphate) and polymer (such as PMMA^15,16^).

For soft glasses, the billet extrusion rig has also been combined with fibre drawing by directly placing the rig on the top of a fiber drawing tower. In this case, the extruded preform is heated by the tower furnace and pulled to reduced diameter^17^. Extrusion of multimaterial preforms is also possible when the billet is formed by a stack of different materials^18^.

Extrusion dies are typically CNC machined, and stainless steel is the most common die material. Recently it was shown that 3D metal printed Cr-Co-Mo and titanium dies are suitable to withstand the high temperature (560–600 °C) and high force (20 kN) involved in the extrusion process of commercial lead-silicate glass^19^ without any mechanical failure in the 3D printed part, opening up unprecedented freedom in die design via 3D printing. More recently^20^, a 3D printed titanium die was used to manufacture a multi-core MOF for imaging purposes. While in^19^ the fiber preform was extruded through the die and subsequently drawn to optical fiber, in^20^ fiber canes with four cores were extruded, then stacked forming a 100 cores structure and finally drawn to fiber. It is important to note that 3D printed dies present a higher surface roughness when compared with machined ones, which can impact the extruded preform and fiber superficial quality. This can be particularly detrimental for the scattering loss of microstructured optical fibers depending, e.g., on the fiber material refractive index and core size. It was shown, however, that polishing just the last few millimeters of the internal surfaces of the die exit (that are accessible via the nozzle end tip) will solve the problem^19^ bringing the surface quality of the extruded optical samples similar to the ones produces with machined dies.

It should be noted that extrusion allows all cross-sectional features to be produced simultaneously, different from the stack-and-draw and drilling procedures where the holes are formed sequentially. The standard way, however, is to extrude the macroscopic preform that is subsequently pulled to the optical fiber stage, meaning a multi-stage process requiring sophisticated equipment.

Standard, all-solid, polymer fibers can be extruded in a single process where two materials are simultaneously fed, forming the fiber with two different materials/compositions to allow the core-cladding interface. Here the raw material can be either polymer pellets or purified monomers.

On the other hand, when glass or polymer MOFs are extruded, the raw material is a bulky billet instead of being in pellets form. The billet is usually cut from a larger body of material or prepared by fusing the raw materials/pellets together completely to ensure high optical quality.

In the last few years, a completely new method to fabricate optical fibers preforms was developed based on the use of additive manufacturing processes. In this case, the preform itself is 3D printed, being subsequently drawn to optical fiber using a dedicated optical fiber drawing tower. Hollow core^21,22^ and solid core^23^ fibers were produced in this way using commercially available polymer filaments. Guidance in the visible and infrared was shown. Recently the technique was expanded to print glass samples with, e.g., borosilicate^24^, silica^25^, and chalcogenide^26^. Step-index optical fibers^27^ were also produced via additive manufacturing in a multi-stage procedure.

Another disruptive development in this area was the idea of simplifying the procedure to manufacture MOFs by extruding it directly from a 3D printer using an acrylonitrile butadiene styrene (ABS) filament and a special, micromachined nozzle^28^. A suspended core fiber was successfully manufactured, but just short lengths of fiber could be obtained before the holes collapsed. Despite this method combined extrusion and pulling in a continuous process, the starting material, i.e., the polymer filament, was prepared from pellets in a separate process with different equipment.

In this work, a single continuous process from pellets to final MOF is achieved by simultaneous extruding pellets and pulling the extruded material, using a compact tabletop, horizontal, pellet-based extruder, originally aimed to produce filaments for 3D printers. This process is fundamentally different from current billet extrusion techniques used to manufacture MOFs, where multiple steps and equipment are involved, including forming a billet using a glass/polymer melting capability, extruding the billet to preform using a ram extruder, and finally drawing the preform to fiber using a drawing tower. By contrast, our new single continuous process technique requires only one basic equipment to go directly from pellets to a MOF. Furthermore, our technique is also different from using the extruder of a 3D printer as the equipment, where the MOF is made from filaments via simultaneous extrusion and pulling^28^, whereas our simultaneous extrusion and pulling process uses pellets directly as starting material, thereby omitting the step of making a filament from pellets via extrusion. In addition, the use of a compact filament extruder to make a fiber directly from pellets allows improved process control and stability as such an extruder is already designed to produce fiber-like structures (i.e., 3D printing filaments). Our process permits the fabrication of complex fiber geometries while being cost, time, energy, and floor space-efficient. 3D metal printing is shown to be a powerful method to manufacture the nozzles employed for the MOF fabrication. Three well established MOF geometries were chosen to demonstrate the viability of our new technique to manufacture a broad range of MOF structures. The extruder flow parameters were determined and the geometry of the fibers was characterized. The optical guidance of a hollow-core fiber was analyzed, showing guidance is in the air core in a spectral range where the fiber material is opaque.

## Polymeric materials: Zeonex and functionalized ABS

Two different thermoplastic polymers were used in this work to fabricate the MOFs - Zeonex, and ABS.

While PMMA is widely used to manufacture standard optical fibers and optical components, cyclic olefin copolymers (COCs)^29^ are increasingly being applied to produce optical waveguides, including specialty optical fibers. The most common COCs for optical fibers are Zeonex and Topas. Their main advantages are the smaller water absorption, less brittle nature, easier moldability, smaller birefringence, higher chemical and thermal stability^30^, and higher transmittance in specific frequency ranges, like the THz^30–32^. Zeonex is also thermally compatible with PMMA making it possible to draw fibers formed of both materials^33^. In this work, Zeonex 480 R grade commercially available^34^ pellets were used. The material has a glass transition temperature (Tg) of 138 °C, a water absorption smaller than 0.01%, and a refractive index (at 589 nm) of 1.525.

ABS was also used to show the possibility of fabricating a doped MOF directly from a functionalized polymeric material. ABS is widely used for injection molding and extrusion in additive manufacturing. It can become whitish under stress due to crazing formation and recover after heating, a characteristic that can be explored in fiber optics sensing applications^35^. ABS is also highly soluble in acetone, allowing preparation of doped ABS powder via adding the dopant material to a diluted ABS solution, followed by evaporation of the solvent. For the proof-of-concept in this work, Rhodamine dye was used as dopant material to fabricate a luminescent MOF. The details of the preparation of the doped ABS powder are described in Methods. Note that PMMA could follow precisely the same doping procedure as it also has a high solubility in acetone.

## Selection of MOF structures

In order to show the versatility of the technique developed along with this work, three different MOF types with distinct cross-sections and geometrical features were selected.

The photonic crystal fiber (PCF) geometry is based on the well-known and widely explored, triangular matrix of air holes and a missing hole (defect) forming the core in the center. The optical properties of PCFs, such as modal area, confinement loss, chromatic dispersion, and birefringence can be widely tuned by adjusting the hole diameter (d) to holes pitch (Λ) ratio^36^. An endless single-mode fiber^3^ that guides just the fundamental mode can, for example, be obtained if the fiber holes are small enough (d/Λ < 0.43). In this work, PCF with a target d/Λ ratio of 0.5 was selected.

The suspended core fiber (SCF) geometry is a widely studied and applied MOF type as it combines a simple design^37^ of a single material fiber with the presence of large air holes around the core area (which is different, for example, to the triangular lattice PCF geometry). This makes the SCF an attractive platform for devices based on the interaction of the optical mode evanescent field and fluid or film of interest^9,38^. In this work, an SCF geometry with three holes was selected.

Hollow core fibers (HCFs) allow the guidance in a low index core due to photonic bandgap or inhibited coupling/antiresonance guidance. These structures present enormous advances in how an optical field can be manipulated and open up a whole range of interesting fundamental and applied scientific opportunities. Fiber-based components are being extensively studied in the last two decades, from broadband, supercontinuum sources to pulse compressors, or high energy all-optical delivery channels. Gas-filled hollow core fibers are a remarkable platform for gas-based nonlinear optics^39,40^. New fiber designs are being extensively studied to broaden the transmittance window, reduce the loss or simplify the waveguide cross-section geometry. Recently, a HCF geometry based on a single ring hexagon-shaped core linked to the fiber cladding via thin struts was proposed and demonstrated in lead-silicate glass. The fiber was manufactured via a billet extrusion, and Raman sensing was shown^41,42^. A similar geometry with slightly more complex cladding structure, and whose fabrication was based on capillary stacking, was subsequently demonstrated in silica^43,44^. In this work, a HCF geometry with single ring hexagon-shaped core was selected.

## Nozzle/die design and manufacturing, and the fiber extruder

A commercial horizontal filament extruder (Filabot EX-2 model) was employed to fabricate three different MOFs. This extruder is initially designed for making polymer filaments (for use in fused deposition modeling 3D printers) by feeding polymer powder or small pellets into a nozzle with circular orifice. By using customized, in-house made nozzles and pulling the material emerging from the nozzle, MOFs with different geometries were made in one continuous process from pellets to fiber.

**Figure 1:**
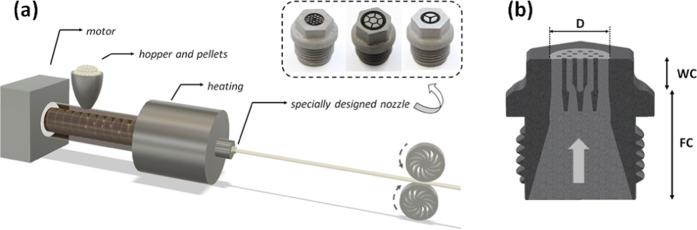
(**a**) Schematic of the fiber extrusion setup with a commercial filament extruder, a filament pulling system, and a 3D printed specially designed nozzle. Inset shows images of the fabricated 3D printed nozzles, from left to right, a two-ring photonic crystal fiber, a hollow-core fiber, and a suspended core fiber. (**b**) plane section view showing the polymer flow (arrow in clear grey) through the feeding chamber (FC) and the welding chamber (WC).

The commercial horizontal filament extruder is sketched in Fig. 1a and is based on a rotating screw that is heated while being fed with the polymer to be extruded. The material should be in the form of pellets or powder. The temperature of the heater around the end of the rotating screw connected to the nozzle is adjusted to match the polymer processing temperature. The polymer is also heated due to the shear exerted by the screw rotation^45^. The material feeding rate is controlled by the speed of the screw rotation. The two puller wheels of the spooler system, which is originally designed to pull and spool the extruded filament, is used to draw the MOF directly from the material exiting the nozzle. The pulling rate, and consequently the fiber diameter, is controlled by adjusting the speed of the puller wheels.

The design of the internal structure of the nozzles is based on a previously developed extrusion die concept^14^. The nozzle design comprises two segments in the direction of the material flow. The first segment, the so-called feeding chamber, contains an array of longitudinal holes to feed the material delivered by the rotating screw into the second segment, the so-called welding chamber, which contains an array of solid longitudinal pins to block the material flow. The obstruction of the flow in the welding chamber results in the formation of the air holes in the material emerging from the welding chamber at the nozzle exit plane into free space. Pulling the material as it exits the nozzle leads finally to the holes in the fiber. The manufacturing process of the nozzles is described in the Methods section.

The inset in Fig. 1a shows photographs of the three types of nozzles used for manufacturing the three types of MOFs in this work: solid core PCF with two rings of holes, HCF, and suspended core fiber SCF. For the sake of clarity, the types of nozzles will be referred to as “PCF nozzle” (nozzle exit diameter, *D* = 13 mm), “HCF nozzle” (*D* = 12.2 mm), and “SCF nozzle” (*D* = 9 mm). Nozzles with more cross-sectional features (like the PCF one) were made a bit wider to avoid the pins to be too thin.

For the three nozzle types (PCF, HCF, SCF), the cross-sectional area, *A*_*F*_, of the flow channels at the nozzle exit plane (from where the material emerges into free space) is 124, 66, and 41 mm^2^, respectively. The flow area fraction, defined as the ratio of the flow channel area, *A*_*F*_, to the total area of the nozzle exit plane, *A*_*T*_ = π/4×*D*^2^, is 93, 56, and 35%, respectively. *A*_*F*_ determines the material feeding rate as described below. The difference of the flow channel area fraction to 100% is the flow obstruction area fraction *A*_*O*_*/A*_*T*_, with *A*_*F*_ + *A*_*O*_ = *A*_*T*_. For the three nozzle types, the *A*_*O*_/*A*_*T*_ values are 7, 44, and 65%, respectively. Note that the solid area, *A*_*solid*_, and the air-filling area, *A*_*air*_, of the material cross-section exiting the nozzle equals the flow channel area (*A*_*solid*_ = *A*_*F*_) and flow obstruction area (*A*_*air*_ = *A*_*O*_) at the nozzle exit, respectively.

Pellet extrusion occurs at a viscosity where the material is molten (viscosity of ~10^0^ dPa.s). Pulling the extruded material (i.e. the material emerging from the nozzle) to a fiber reduces the cross-sectional area of the material and also avoids the holes to collapse. This is fundamentally different from a bulk billet extrusion that occurs at a higher viscosity where the material is only softened (viscosity of ~10^8^ dPa.s), and therefore no drawing force is necessary to keep the air holes open.

The extruded mass flow rate (*μ*_*feed*_ = mass/time) was measured for the PCF and HCF nozzles to calculate the expected fiber diameter for a certain pulling rate. Zeonex polymer pellets were freely extruded without being pulled, while the speed of the screw rotation was adjusted via a knob on the extruder body. For each knob position, five extruded samples were obtained, and their mass measured. The extrusion time (in the 20-90 s range) was chosen for each position to allow each sample mass to be around 1.5 to 3.5 g. The mass flow rate of a certain knob position is the average of the mass flow rates of all the samples made at this knob position.

**Figure 2:**
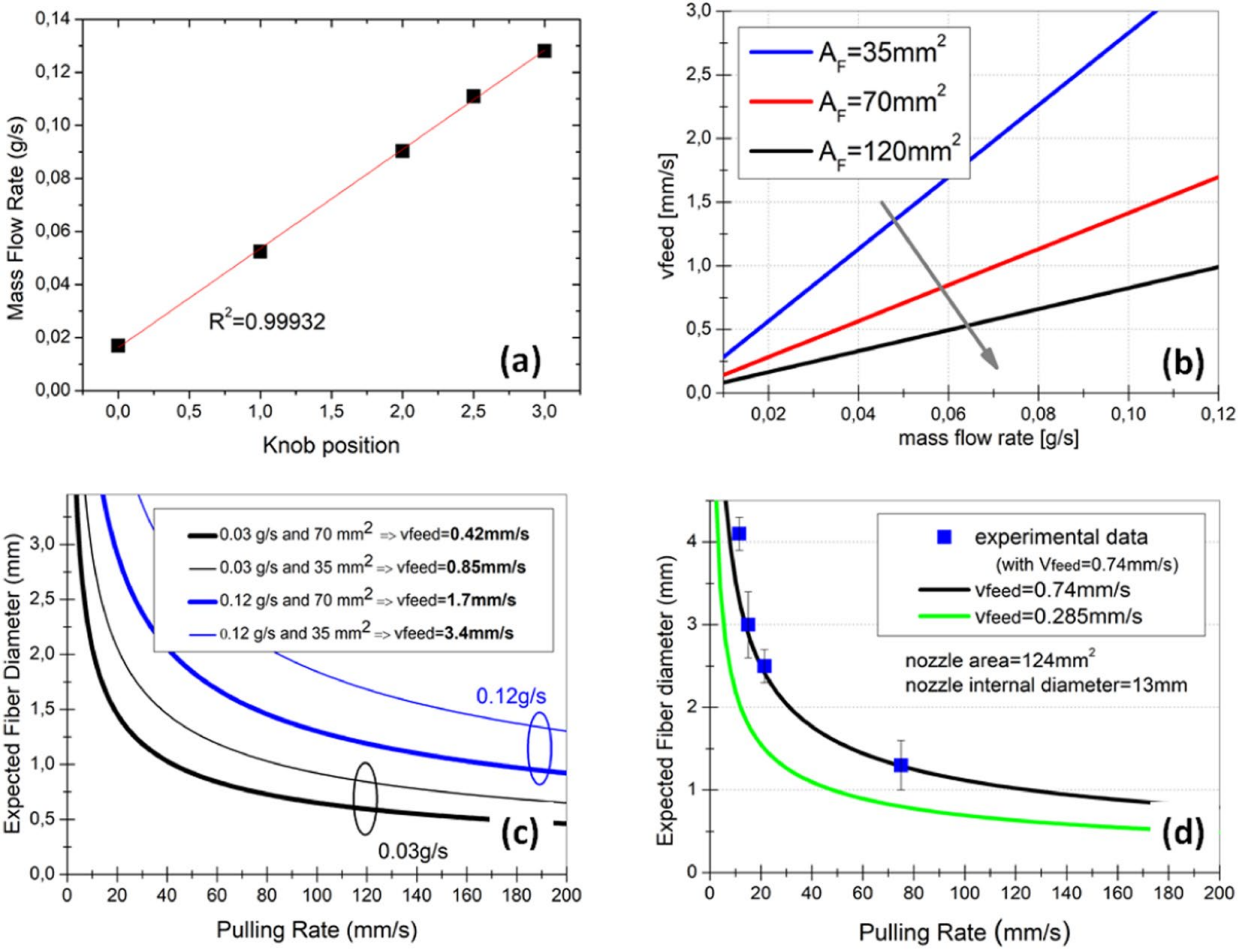
(**a**) Measured extruder mass flow rate using Zeonex and “PCF nozzle” and “HCF nozzle”; (**b**) calculated feeding rate for different flow channel areas (A_F_) at the nozzle exit plane; (**c**) calculated expected final fiber diameter for internal nozzle diameter of 10 mm, mass flow rates of 0.03 and 0.12 g/s and nozzle die exit area of 35 and 70 mm^2^; (**d**) calculated expected fiber diameter when using the “PCF nozzle” (D = 13 mm, A = 124mm^2^) for two different feeding rates (0.285 and 0.74 mms), and experimentally measured diameters when using a feeding rate of 0.74 mm/s.

Figure 2a shows the mass flow rate in the range of 0.02 to 0.13 g/s increases linearly with the knob position (fitting slope = 0.0374), which is a measure for the screw rotation speed, for Zeonex extruded at 208 °C with the PCF and HCF nozzles. No noticeable difference was found for 215 and 222 °C extrusion temperature, indicating the screw rotation speed is the determining factor for the mass flow rate.

With the experimentally obtained mass flow rate (*μ*_*feed*_ = 0.02–0.13 g/s), the known polymer density (*ρ* = 1.01 g/cm^3^ for Zeonex), and the known flow channel area at the nozzle exit (*A*_*F*_) the polymer feeding rate (*v*_*feed*_) can be calculated using Eq. (1),

1 $${v}_{feed}=\frac{{\mu }_{feed}}{\rho \cdot {A}_{F}}$$

For different knob positions (0 to 3) and typical values of nozzle flow channel area (*A*_*F*_ = 35, 70 and 120 mm^2^), the calculated feeding rate is in the range of *v*_*feed*_ = 0.2 to 3.0 mm/s. Figure 2b shows that a larger *A*_*F*_ value leads to a smaller feeding rate for the same mass flow rate, i.e. for the same screw speed.

The extruded polymer material exiting the nozzles can be pulled manually or with the pullers shown in Fig. 1. Assuming the air filling fraction in the fiber cross-section is maintained relative to that at the nozzle exit as the material is being pulled, the mass conservation law allows calculation of the fiber diameter (*d*) from the known values of nozzle exit diameter (*D*), feeding rate (*v*_*feed*_), and the fiber pulling rate (*v*_*pull*_).

2 $$d=D\sqrt{\frac{{v}_{feed}}{{v}_{pull}}}=\sqrt{\frac{{\mu }_{feed}}{\rho \cdot {A}_{F}\cdot {v}_{pull}}}$$

For typical values of *D* (10 mm), *μ*_*feed*_ (0.03 and 0.12 g/s), *A*_*F*_ (35 and 70 mm^2^), and *ρ* of Zeonex, Fig. 2c shows the calculated fiber diameter is <2 mm for pulling rates of 10-100 mm/s (i.e. 0.6–6 m/min) and feeding rates of 0.4–3.4 mm/s (i.e. 25–204 mm/min). For a fixed pulling rate, the fiber diameter decreases with decreasing mass flow rates and increasing the flow channel area of the nozzle exit. In other words, it is easier to obtain a thinner fiber for slow feeding rate, large solid-filling fraction of the fiber (i.e. small air filling fraction), small nozzle exit diameter and fast fiber pulling rate.

It is interesting to note that for pulling an optical fiber with a pellet-based extruder (this work), the typical feeding rate (~ 10–100 mm/min) is 1–2 orders of magnitude higher than the values used with a standard fiber drawing tower (~mm/min). The main consequence is that, to obtain a fiber with similar diameter, a higher pulling rate is necessary with an extruder compared with a drawing tower due to the faster feeding rate of the extruder. The main reason is the screw rotation control set by the extruder manufacturer originally designed for high throughput production of filaments and hence high mass flow rates.

Figure 2d presents the calculated fiber diameter as a function of the pulling rate for the “PCF nozzle” design (*D* = 13 mm and *A*_*F*_ = 124mm^2^) with two different feeding rates: *v*_*feed*_ of 0.285 mm/s (knob position of 0.5, mass flow rate of 0.035 g/s) and 0.74 mm/s (knob position of 2.0, mass flow rate of 0.09 g/s). Fibers were produced with the 0.74 mm/s feeding rate, and their diameter manually characterized by a caliper. A good agreement between the calculated and measured fiber diameters is obtained (Fig. 2d).

## Fabrication of characterization of MOFs

All manufactured fibers were hand cleaved with new razor blades without any procedure optimization. It is noteworthy that heating the fiber and/or the razor blade^46^ or polishing the fiber end face^47^ after cleaving could enhance the cross-section smoothness.

### Solid core PCF

A Zeonex solid core PCF with two rings of holes was produced using the nozzle shown in the inset of Fig. 1a (left picture). The 3D printed nozzle has 18 pins in its welding chamber to allow the air hole pattern to be formed. These pins are 0.8 mm thick and 1.55 mm away from each other, leading to a d/Λ ratio of 0.52. The overall internal diameter is 13 mm.

**Figure 3:**
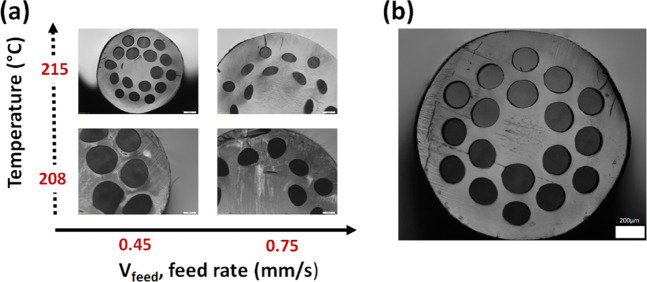
(**a**) Optical images of the Zeonex polymer solid core PCFs made at different temperatures and different feeding rate. All fibers were pulled at 20–50 mm/s and have an external diameter between 1.5 and 2.0 mm. (**b**) 1450 µm diameter fiber with 385 µm core diameter. The white scale bars indicate 200 µm.

Figure 3a shows the cross-section of fibers extruded at two different temperatures (208 and 215 °C) and two different feeding rates (0.45 and 0.75 mm/s) using a pulling rate around 20–50 mm/s. For higher temperatures and faster feeding rates, the cladding air filling fraction (d/Λ) is substantially reduced due the partial collapse of the fiber cross-section that is being pulled at low viscosity. Figure 3b shows one fiber exemple with an external diameter of 1450 µm, average holes diameter of 200 µm and an average pitch of 270 µm (d/Λ~ 0.75).

As the solid core PCF geometry has a large flow channel area (*A*_*F*_) in the nozzle design, and the printed nozzle a big diameter (*D*), to further reduce the fiber diameter some modifications in the extruder would be necessary. In particular, a higher pulling rate would help obtaining thinner fibers with this geometry.

### Suspended core fiber (SCF)

**Figure 4:**
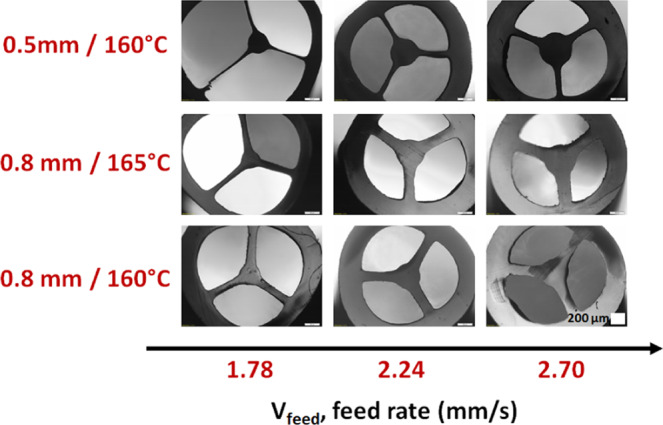
Optical images of ABS polymer SCFs extruded at 160 and 165 °C with different feeding rates and using two “SCF nozzles” with different strut thickness (0.5 and 0.8 mm). Fiber cracks are just superficial and due to unoptimized cleaving procedure. The white scale bar on the bottom right image is 200 μm, and the same for all nine images.

Two 3D printed suspended-core nozzles, whose unique difference is the thickness of the strut (0.5 or 0.8 mm), were used with transparent ABS pellets to manufacture polymeric suspended core fibers. To optimize the fabrication parameters, the extruder screw speed was varied in order to adjust the feeding rate from 1.5 to 3 mm/s, and the results are summarized in Fig. 4. The pulling rate was adjusted to produce fibers with similar diameters. To be able to vary the feeding rate, while maintaining the fiber diameter and not exceeding the maximum pulling rate, fibers with 2.0 mm diameter were manufactured.

The results show that the fiber strut thickness increases with increasing feeding rate. Moreover, as expected, the nozzle with thicker struts (middle and bottom rows) generates fibers with thicker struts.

In order to keep the confinement loss as low as possible, fibers with thinner struts are desired. From the results shown in Fig. 4, the ideal conditions are low feeding rate, temperature around 160 °C, and the use of the nozzle with 0.5 mm wide struts.

**Figure 5:**
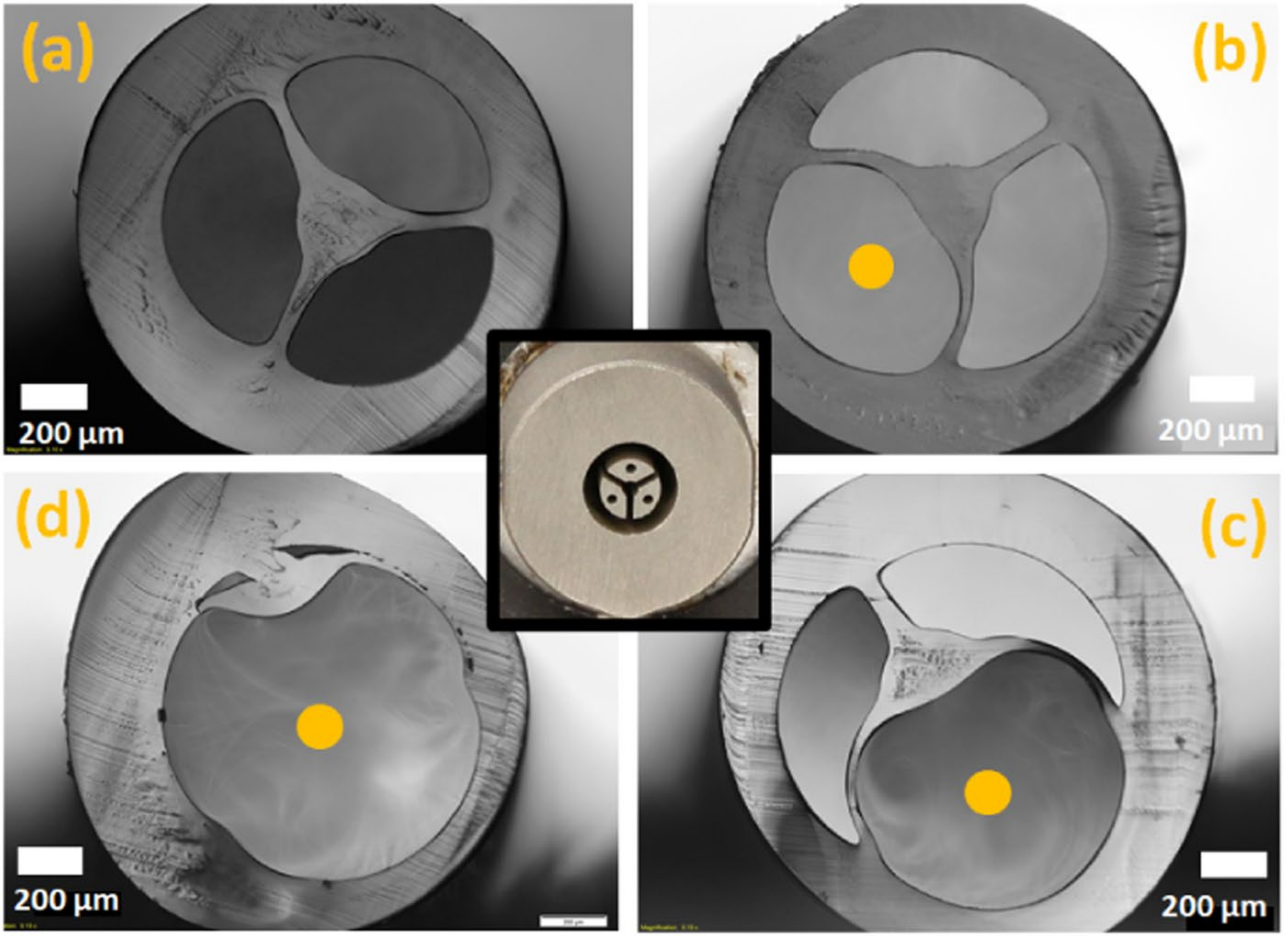
The center of the figure shows a specially designed nozzle to control the internal holes pressure during extrusion. Optical images show a Zeonex polymer SCF with an external diameter of 1500 µm fabricated without applied pressure (**a**) and with increased pressure (from **b**–**d**). The hole with positive pressure is indicated with a yellow dot. The white scale bars indicate 200 µm.

MOF fabrication usually involves the use of positive or negative pressure in the preform to control the holes size (and shape) during the pulling process. In this work, this concept was explored during the fiber pellets extrusion. A special die was developed, similar to the one shown in the inset of Fig. 1a (right picture), but with air channels in the flow obstruction regions (picture in the center of Fig. 5). As a proof-of-concept experiment, one of the holes was pressurized during the extrusion of the Zeonex pellets. The pressure was increased during the fiber drawing to show the enlargement of one of the holes and, in consequence, the core´s displacement from the center to the outer solid region. The temperature was kept constant (at 234 °C) during the whole process. Optical images of 1500 µm thick fibers are shown in Fig. 5 with increased pressure from 0 mbar (Fig. 5a) to 20, 40 and 80 mbar (Fig. 5b,c,d). As expected, the struts around the expanded hole become thinner.

**Figure 6:**
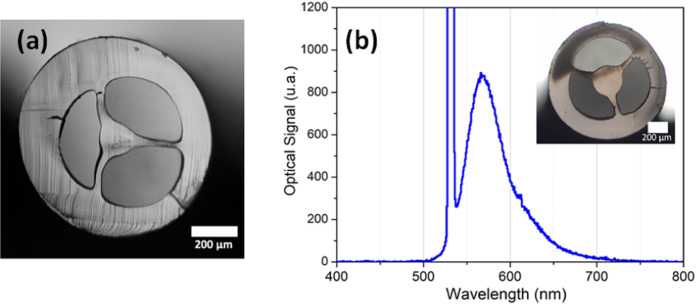
(**a**) Optical image of 835 µm diameter Zeonex polymer SCF; (**b**) Luminescence signal measured when a Rhodamine B-doped ABS SCF (inset) is excited with a 532 nm laser.

An 835 µm diameter Zeonex fiber with 130 µm diameter core was also manufactured at 240 °C and at low feeding rate (Fig. 6a). The fiber is several meters long without any significant geometrical deformation. Using a cutback procedure and a He-Ne laser at 633 nm, coupled in the core of a 30 cm long fiber, the loss of the Zeonex SCF was measured to be 25 dB/m. Zeonex material loss is expected to be around few dB per meter^32^ in the visible range. The higher loss in our result is expected to be related to the lack of thermal treatment in the pellets before the extrusion, a problem already faced by other groups^32,48^.

To demonstrate the possibility to readily manufacture a doped MOF with the technique summarized in Fig. 9 (Methods), a Rhodamine B doped ABS fiber was extruded. Here 40 grams of ABS were dissolved in 250 ml of acetone, and 6 ml of Rhodamine B with 1,05*10^−3^ mol/l concentration were added to the solution. The produced powder was extruded at 160 °C with a low feeding rate. The final fiber has an external diameter of 1200 µm and a core diameter of 300 µm. To confirm the luminescence of the dye-doped into the fiber material, a 532 nm CW laser was coupled to 3 cm long fiber with the help of a 10x objective lens. The fluorescence signal was collected from the lateral surface of the fiber with a handheld spectrometer (Ocean Optics QE Pro). Figure 6b shows, together with the fiber cross-section, the dye luminescence centered at 570 nm and the pump laser peak at 532 nm. This proof-of-concept experiment shows the ability to produce doped microstructured optical fibers easily.

### Hollow core fiber (HCF)

**Figure 7:**
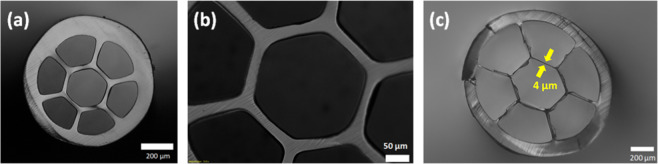
(**a,b**) Optical images of Zeonex polymer HCFs with external diameter of 800 µm, core size of 230 and core ring thickness of 21 µm; (**c**) Fiber with 4 µm core ring thickness and core diameter of 290 µm.

HCFs with six struts suspending the hollow core were made using 3D printed nozzles (internal diameter of 12.2 mm) like the one shown in the inset of Fig. 1a (middle picture). The fibers were fabricated using an extrusion temperature of 220 °C. Figure 7 shows optical images of HCFs made using a nozzle with 0.8 mm (Fig. 7a,b) and 0.5 mm (Fig. 7c) struts. The fiber shown in Fig. 7a,b has an external diameter of 800 µm, core size of 230 µm and a core ring thickness of 21 µm. When using a nozzle with thinner struts, fibers with a thinner core ring are obtained (Fig. 7c). Here some struts are broken or are not straight due to the fiber cleaving process.

**Figure 8:**
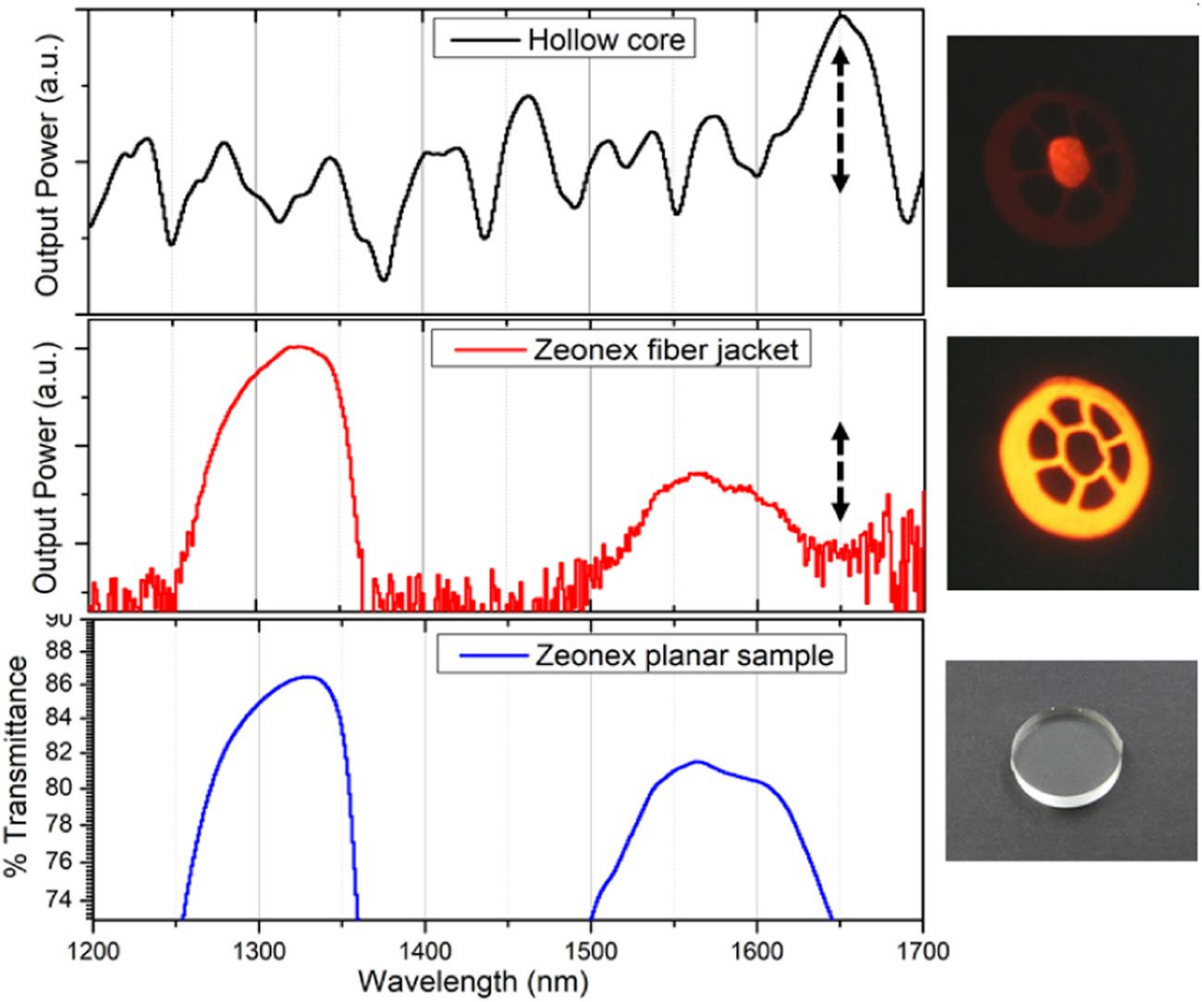
Transmittance of a Zeonex polymer HCF with core ring thickness and diameter of 32 and 300 µm, respectively. The top figure shows the spectrum when light is coupled into the hollow core, while the central figure shows the case when light is coupled in the outer solid region. The arrows at 1650 nm indicate 10 dB, and the images on the right are the far-field images of each case. The bottom figure presents the transmittance spectrum (in logarithmic scale) of a planar Zeonex sample with 6.2 mm thickness, and the image on the right is a photograph of the sample.

The fiber transmittance was measured for an HCF with an external diameter of 1090 µm, core size of 300 µm, strut thickness of 32 µm, and length of 23 cm using a supercontinuum broadband source (NKT Photonic SuperK Extreme) and a 4x objective lens for launching the light into the fiber. The light was coupled to the hollow core (Fig. 8, top) or to the solid outer region (Fig. 8, middle). In both cases, the fiber end face image was taken with a 10x objective lens and directed to the optical port of an optical spectrum analyzer. The recorded spectra are presented in Fig. 8.

To determine the material transmittance, a 6.2 mm thick Zeonex disc was measured using a UV-vis spectrometer (Cary 5000). The transmittance in the wavelength range used for fiber characterization (1200–1700 nm) is presented in Fig. 8 (bottom) and shows the same profile as the outer solid region of the fiber (Fig. 8, middle). High transmittance of 86% is observed at 1330 nm, which represents an absorption coefficient of 0.234 cm^−1^. At 1360 and 1646 nm the transmittance is 73%, corresponding to an absorption coefficient of 0.508 cm^−1^. According to these data, the transmittance difference from 1330 to 1360 nm is 27 dB for a 23 cm long fiber sample, in excellent agreement with the transmittance measured for the outer solid region of the fiber (Fig. 8, middle).

Due to the multimode characteristic of the fiber in the measured wavelength range of 1200–1700 nm, and the thick (32 µm) struts forming the core, the antiresonance transmittance spectrum signature is not as apparent as for a single-mode waveguide. Nevertheless, noticeable transmittance peaks are observed when light is coupled into the air core. Of particular note is the >10dB peak at 1650 nm (shown by the dotted arrow with length of 10 dB in Fig. 8a). The fiber material itself is highly lossy at this wavelength, as shown by the spectrum when light is launched into the solid outer region of the fiber and by the spectrum of the Zeonex disc. The high transmittance in the hollow core despite high material loss demonstrates the air core guidance.

## Conclusions

A new ultra-simplified optical fiber fabrication procedure is demonstrated. Instead of requiring multiple stages using different and sophisticated equipment (such as an expensive optical fiber drawing tower), just a low-cost off-the-shelf pellet-based filament extruder is required. No preform fabrication is needed, meaning that drilling, capillary stacking, billet extrusion, or casting are not necessary to make the preform for fiber drawing. Therefore, our fiber fabrication procedure is time-efficient, and a MOF can be produced in less than 30 minutes. The process is also highly efficient in terms of electrical power and used floor-space, making straight-forward fabricating fibers in confined places and small laboratories with limited resources.

The produced fiber total length is no longer restricted by the preform or billet dimensions but just on how long the extruder can run continuously. As the pellets could potentially be fed to the extruder hopper continuously, the limitation is just related to how long the extruder could run uninterruptedly. At an average feeding rate (0.06 g/s, see Fig. 2a), for example, one kilometer of 500 µm diameter fiber could be produced in an hour.

Apart from the virtually unlimited fiber length, the process is also versatile, allowing the quick production of a variety of different fiber geometries by merely changing the nozzle. The developed process opens the possibility to produce customized fibers on-demand, and with fast structural optimization to a specific target application.

3D printed titanium nozzles were used to fabricate three different types of specially designed MOFs. This demonstrates the flexibility of our new technique to manufacture different fiber designs.

As a proof-of-concept of the ability to make functionalized fibers with our new technique, a Rhodamine B doped polymer SCF was demonstrated. In the future, doping with other materials of optical interest such as quantum dots, or nanoparticles, will be explored.

For a fabricated HCF with hexagon-shaped core and single ring cladding, guidance in the near-IR spectral range, where the fiber material has high loss, was observed. The excellent quality of the HCF cross-section geometry quality, e.g., the struts uniformity, is remarkable for a polymer HCF considering the easy route followed for its fabrication. The obtained high geometrical quality combined with a hollow core geometry, where the material loss has a small influence on light guidance, opens up the possibility to explore this platform for mid-IR or THz guidance^22,49^. A Zeonex HCF as-fabricated here is of particular interest for THz guidance due to the low loss of Zeonex in the THz frequency range^31^. In the future, negative curvature HCFs produced with our new technique will be investigated with the goal to reduce the fiber loss via smaller overlap between the optical mode and the fiber material. Recent developments already demonstrated – using vertical extruders - the feasibility to extrude negative curvature hollow-core fibers with specially designed nozzles^50,51^.

The possibility to pressurize the internal structure of the fiber in real-time during its fabrication was shown. This is, to the best of our knowledge, the first time a MOF is extruded with the possibility of real-time pressurization. This mimics the existing fabrication degree of freedom of an optical fiber drawing tower. For billet extrusion, such pressurization is not available, making it challenging to control hole size beyond selecting the size of the flow obstructions.

This work demonstrates a new route to easily fabricate specially designed optical fibers. There is room for overall optimization, including the extrusion parameters such as screw profile, different hot zones temperatures along the screw, and pellets thermal treatment before the extrusion starts. The nozzle design can also be ophmized to, for example, enhance the material flow to the jacket section of the fiber leading to more robust structures.

The ability to draw the fiber faster would, for example, simplify the process of obtaining thin fibers, which are made using nozzles with large exit area such as “solid core PCF” nozzles used here. A fiber tractor pulling system, e.g., would allow faster pulling rates with a smoother operation due to the large contact area between the pulling structure and the fiber ifself.

While this work focuses on polymer optical fibers, future progresses include the possibility to fabricate glass MOFs, following recent developments with 3D printing glassy materials.

## Methods

### Nozzle fabrication

The nozzles were manufactured using 3D metal printer based on selective laser melting of titanium alloy (90% titanium) powder, with 85% of the particles having a diameter smaller than 35 µm. The 3D metal printer uses a 300 W, 1070 nm fiber laser to melt the metal particles, making them fuse together and thus forming the metal structure layer-by-layer with a 30 µm resolution. The 3D printing time for a nozzle is around 3 hours. To release the built-in stress, the as-printed nozzle was heat-treated at 650 °C for two hours. It should be noted that each nozzle can be used multiple times, probably hundreds of times.

Each nozzle has a cylindrical outer shape with a typical length and diameter in the 20–25 mm range. The polymer entrance side has an external thread to screw it into the filament extruder. The polymer exit side has a sophisticated internal structure, from which the optical fiber emerges into free space.

The internal construction of the nozzle follows the same design concept of extrusion dies used to manufacture optical fiber preforms in a vertical billet extruder^14^. It includes an initial feeding chamber that directs the flow of material towards the welding chamber, where the material emerging from the feed holes fuse together into a single body of material. Solid obstructions (running parallel to the nozzle axis) in the welding chamber prevent the material flowing to these spaces, thereby forming holes in the fused material when exiting the nozzle. Figure 1b shows a lateral cut of one of the nozzles showing the polymer flow in light grey with the feeding and welding chambers indicated.

### Polymer functionalization

Initially, a commercial 1.75 mm thick 3D printed ABS filament was cut in 3–4 cm long sections, immersed in acetone (20 grams of ABS per 125 ml acetone), and stirred for 2 hours with a lid over the beaker. This allows the formation of a homogeneous dissolved ABS “*soup*”.

The next step consists of adding the doping material and stirring an extra 30 min after which the lid is removed. With the lid off, the solvent starts to evaporate, and the solution becomes thicker. After 90 minutes, the soup is poured in Petri dishes and let it dry overnight. The dry films can easily be removed from the plates and fragmented in few pieces.

**Figure 9:**
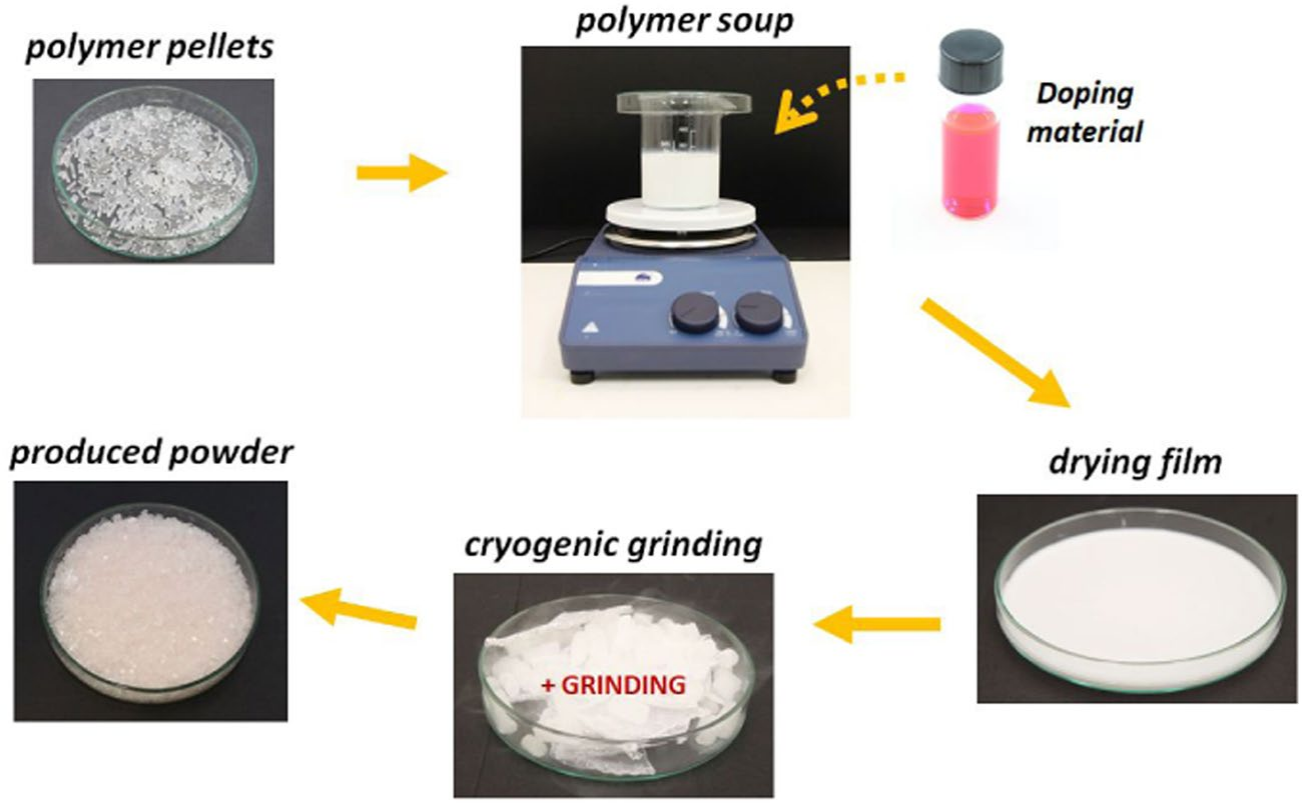
functionalizing polymer process overview that starts with pieces of the chosen polymer and finalizes with a polymer powder mixed with the desired material.

The last step is cryogenic grinding^52^ the film to make fine polymer powder to be used in the filament extruder. The process involves the use of dry ice (−78 °C) in a 2:1 mass ratio with ABS to cool down the polymer while this is being ground. The dry ice avoids overheating that would, otherwise, melt the plastic, and sublimes to carbon dioxide. The overall procedure to produce the doped ABS powder that will be used to feed the filament extruder is summarized in Fig. 9.
